# Spirituality and behavioural addictions: narrative review

**DOI:** 10.1192/bji.2024.9

**Published:** 2024-08

**Authors:** Mitika Kanabar, Preethy Kathiresan, Hussien Elkholy, Arash Khojasteh Zonoozi, Laura Orsolini, Jiang Long, Mehdi Farokhnia, Roshan Bhad, Jenna L. Butner, Francina Fonseca, Vicky Phan, Sophia Achab, Marc N. Potenza

**Affiliations:** 1 Physician Director of Education, Southern California Permanente Medical Group, Lancaster, California, USA; 2 Department of Psychiatry, All India Institute of Medical Sciences, New Delhi, India.; 3 Professor of Psychiatry, Department of Neurology and Psychiatry, Faculty of Medicine, Ain Shams University, Cairo, Egypt; 4 Iranian National Center for Addiction Studies, Tehran University of Medical Sciences, Tehran, Iran; 5 Assistant Professor of Psychiatry, Unit of Clinical Psychiatry, Department of Neurosciences/DIMSC, Polytechnic University of Marche, Ancona, Italy; 6 Psychiatrist, Shanghai Mental Health Center, Shanghai Jiao Tong University School of Medicine, Shanghai, China; 7 National Institute on Drug Abuse and National Institute on Alcohol Abuse and Alcoholism, National Institutes of Health, Baltimore, Maryland, USA; 8 National Drug Dependence Treatment Centre, All India Institute of Medical Sciences, New Delhi, India; 9 Assistant Professor Adjunct of Medicine (General Medicine), Department of Internal Medicine, Yale University School of Medicine, New Haven, Connecticut, USA; 10 Head of Addiction Program, Mental Health and Research Institute, Hospital del Mar, Barcelona, Spain; 11 Addiction Psychiatrist, Turning Point Eastern Treatment Services, Melbourne, Australia; 12 Head of Addictive Behaviours, Centre ReConnecte, Addiction Division, Department of Psychiatry, University Hospitals of Geneva, Geneva, Switzerland; 13 Departments of Psychiatry, Child Study Center, and Neuroscience, Yale University School of Medicine, New Haven, Connecticut, USA

**Keywords:** Mental health services, patients and service users, psychosocial interventions, transcultural psychiatry, philosophy

## Abstract

The relationship between spirituality and behavioural addictions is complex. Although some studies have suggested spirituality to be a protective factor helping in recovery from addictive behaviours, others have found spirituality to be a potential risk factor. To better understand the relationship between spirituality and various behavioural addictions, this review summarises the literature on the association between spirituality and the following behavioural addictions: gaming disorder, gambling disorder, problematic internet use, problematic smartphone use, compulsive sexual behaviour disorder and compulsive buying/shopping disorder. Implications for clinical practice and future research are discussed.

The global prevalence of behavioural addictions is estimated at close to 11%.^1^ The current classification systems, namely DSM-5 and ICD-11, include only gambling and gaming disorders as disorders due to addictive behaviours. However, addiction in other areas, including internet use, excessive buying/shopping, pornography use, social media use and binge-watching have also been noted in the literature.

Spirituality has been studied considerably in relation to substance use disorders and associated with recovery. Spirituality can be conceptualised as a distinct, potentially creative and widespread human experience arising from an inner awareness of individuals and within communities, social groups and traditions.

Several traditions and practices have roots in spiritual aspects, including a higher power, existence in the present moment, transcendence and finding happiness. For some, spirituality may equate to religiosity, whereas others may identify as spiritual but not religious. Spirituality is viewed as being central to the 12-step recovery model used, for example, by Alcoholics Anonymous. Spirituality has been associated with decreased drug and alcohol use, but there is a need to synthesise the literature on spirituality and behavioural addictions.

In the USA, groups such as Celebrate Recovery help people understand the 12-step model through Christian scripture. From a Buddhist Dharma standpoint, several groups, such as Refuge Recovery and Buddhist Recovery, deliver online programmes based on Buddhist principles and practices, adapting the 12-step model, to help people with their spiritual needs.

Mindfulness and spirituality are related, as both focus on cultivating enhanced awareness of the present moment, inner exploration and a deeper connection to oneself, others and the universe. Newer models based on mindfulness have been adapted from Zen Buddhism, which itself finds some roots/similarities with Dhyana in Yoga, Hinduism and other Indic religions. Several aspects have been modified to remove the concept of religiousness and operationalise mindfulness alone to make it applicable to a broader audience. Such approaches are outlined in Table 1.

**Table 1:** Mindfulness-based approaches

Approach type	Focus area
Mindfulness-based stress reduction	General principles for stress reduction, caregiver benefit
Mindfulness-based relapse prevention	Combining cognitive and mindfulness skills for responding to stimuli
Mindfulness-based cognitive therapy	Combining cognitive and mindfulness skills for co-occurring mood and addiction problems
Mindfulness-oriented recovery enhancement	Restructuring reward processing for addiction, chronic pain and post-traumatic stress

Yoga, though perceived as a form of exercise, is spiritually oriented. Ashtanga (meaning eight-limbed) yoga is a path for meditation and self-realisation, with rules for responding to stimuli in a balanced, ethical manner and may benefit addressing of behavioural addictions.^2^

Spirituality-based approaches to addiction care help patients by using themes of deep acceptance, compassion, support and forgiveness while teaching new coping skills to adapt to triggers. Recovery capital refers to the internal and external resources available to an individual for promoting sustained recovery from addiction. These resources may be classified into five primary domains: human, social, physical, cultural and community. Recovery capital has shown greatest effects in the adolescent population. Spirituality can help people improve their internal coping skills and find a recovery community. The Buddhism-based group Recovery Dharma has evidence of increasing recovery capital for its participants. Spirituality and the Islamic faith have also been studied with respect to addiction.

Our narrative review aims to give an overview of the literature on spirituality and behavioural addictions. We come from diverse social and cultural backgrounds, and some of us have witnessed first-hand how spirituality and religion are praised by some patients as protective factors in various forms of addiction or as important in their recovery. Also, some of us have received comments on how behavioural addictions have affected the religious and spiritual practices of individuals who engage in them.

## Method

As spirituality and religiosity are intertwined, in the current review, we focused predominantly on articles that specifically mentioned spirituality as a component of either assessment or intervention. Other relevant studies on religiosity and behavioural addictions have been included. Systematic reviews and studies with larger sample sizes have been given preference. The studies are summarised in Table 2.

**Table 2:** Studies on spirituality and behavioural addiction

Type of behavioural addiction	Studies reviewed	Predominant association with spirituality
Gaming disorder	Chinese study, 401 students^3^ European study, 5922 participants^4^	Negative association
Gambling disorder	Swiss study, 5179 youths^5^ English study, 7403 participants^6^ Scoping review, 25 studies^7^	Mixed association
Problematic internet use	Systematic review, 13 studies^8^ Swiss study, 5179 youths^5^	Mixed association
Problematic smartphone use	Korean study, 285 adolescents^9^	Negative association
Compulsive sexual behaviour disorder	Systematic review, 59 studies^11^	Positive association
Compulsive buying/shopping disorder	Polish study, 430 adults^11^	Negative association

## Results

### Spirituality and gaming disorders

The available studies have predominantly found spirituality to be negatively associated with gaming disorder, although there are some inconsistencies.^3,4^

### Spirituality and gambling disorders

The studies on the association between spirituality and gambling disorders have been inconsistent, finding a positive, negative or no association.^5–7^

### Spirituality and problematic internet use

The studies are mixed, some positive and some negative for benefit of religiosity or spirituality in problematic internet use. Among adolescents, there is some evidence that greater spirituality is associated with higher risk of excessive internet use and gambling disorder but lower risk of substance use. Spirituality and religiosity together fared better. We could hypothesise that internet use or gambling may be considered more acceptable than substance use for mood mediation.^5,8^

### Spirituality and problematic smartphone use

Lower spiritual well-being has been correlated with increased likelihood of problematic smartphone use in adolescents; among medical students, psychological capital can moderate the relationship.^9^

### Spirituality and compulsive sexual behaviour disorder

The majority of studies in our sample found a significant relationship between compulsive sexual behaviour and religiosity or spirituality. As almost all studies assessing spirituality had also assessed religiosity, it is difficult to segregate the effect of spirituality alone on compulsive sexual behaviour disorder. It is possible that ‘moral incongruence’ might be a moderating or mediating factor between spirituality and compulsive sexual behaviour/problematic pornography use.^10^

### Spirituality and compulsive buying/shopping disorder

Daily spiritual experiences have been found to be protective, with a negative association with compulsive buying/shopping disorder.^11^

## Discussion and conclusion

There is a paucity of studies that have focused on behavioural addictions and spirituality in general. Although many proponents of spirituality consider it an essential factor that links to greater likelihood of recovery, and there is evidence for this, there is also evidence that points in the opposite direction, especially with respect to compulsive sexual behaviours, and some evidence that suggests no associations.

These variations in the associations could be due to a number of factors. One important reason could be the difference in the spiritual constructs being studied. For example, in the systematic review by Jennings and colleagues, indicators of health spirituality were negatively associated with compulsive sexual behaviour whereas spiritual struggles were positively associated with such behaviour.^10^ In the case of internet addiction, an umbrella construct involving different online activities, the different activities might relate differently to specific spiritual constructs, leading to variations in the associations. Studying each online behaviour/disorder independently might provide greater insight into the relationships with spirituality. Another important factor could be the heterogeneity of the sample included in the studies. Differences in ethnic, cultural and other sociodemographic characteristics could affect associations between spirituality and addictive behaviours.

One main reason for a positive association could be the greater spiritual struggles among patients with behavioural addictions. Positive associations between religious beliefs and compulsive sexual behaviour may additionally be explained by cognitive dissonance between the psychological effects of a desired conduct and a range of strong values and morals condemning these, and this could hold for behavioural and substance addictions broadly. Future studies focusing on interventions that help individuals deal with spiritual struggles may be useful to understand better the links between addictive behaviours and spirituality.

Recovery capital, which is positively associated with spirituality, has been found to be an important factor for recovery from behavioural addictions. Recovery capital can facilitate spirituality by providing supportive social networks and resources conducive to spiritual exploration and growth, fostering resilience and empowerment essential for engaging in meaningful spiritual practices. Future studies could further explore how recovery capital and spirituality may be utilised to help people suffering from behavioural addictions.

The instruments used for assessment in the literature were highly diverse and it is thus difficult to compare results across studies. Also, some of the studies are secondary analyses, and the main objective of the primary studies was often not the study of the association between addictions and spirituality, and for this reason, the measurement approaches used in multiple studies were often not optimal for identifying this association.

Given that treatment and support options for behavioural addictions remain limited, developing resources to support spirituality and overcome spiritual struggles in people experiencing addictions could enhance and support care. More studies are needed that investigate (using consistent validated scales) the relationship between spirituality and behavioural addictions and the role of spirituality in addiction recovery across different social contexts and cultures.

## Data Availability

Data availability is not applicable to this article as no new data were created or analysed in this study.
